# G-Quadruplexes Involving Both Strands of Genomic DNA Are Highly Abundant and Colocalize with Functional Sites in the Human Genome

**DOI:** 10.1371/journal.pone.0146174

**Published:** 2016-01-04

**Authors:** Andrzej S Kudlicki

**Affiliations:** 1 Department of Biochemistry and Molecular Biology, University of Texas Medical Branch, Galveston, Texas, United States of America; 2 Institute for Translational Sciences, University of Texas Medical Branch, Galveston, Texas, United States of America; 3 Sealy Center for Molecular Medicine, University of Texas Medical Branch, Galveston, Texas, United States of America; Tulane University Health Sciences Center, UNITED STATES

## Abstract

The G-quadruplex is a non-canonical DNA structure biologically significant in DNA replication, transcription and telomere stability. To date, only G4s with all guanines originating from the same strand of DNA have been considered in the context of the human nuclear genome. Here, I discuss interstrand topological configurations of G-quadruplex DNA, consisting of guanines from both strands of genomic DNA; an algorithm is presented for predicting such structures. I have identified over 550,000 non-overlapping interstrand G-quadruplex forming sequences in the human genome—significantly more than intrastrand configurations. Functional analysis of interstrand G-quadruplex sites shows strong association with transcription initiation, the results are consistent with the XPB and XPD transcriptional helicases binding only to G-quadruplex DNA with interstrand topology. Interstrand quadruplexes are also enriched in origin of replication sites. Several topology classes of interstrand quadruplex-forming sequences are possible, and different topologies are enriched in different types of structural elements. The list of interstrand quadruplex forming sequences, and the computer program used for their prediction are available at the web address *http*:*//moment*.*utmb*.*edu/allquads*.

## Introduction

The G-quadruplex (G4) is a non-canonical DNA structure consisting of four strands stabilized by Hoogsteen bonds that has received significant attention in the recent years. G4s have been implicated in numerous cellular contexts and functions [1,2], including telomeres [3], *cis*-acting regulatory elements [4], transcription [5], and replication [6,7,8]. Four runs of guanine must be present in the DNA sequence from which a G4 is created [9,10]. While bimolecular or tetramolecular G-quadruplexes have been discussed in the context of short oligomers, or of interchromosomal interactions of telomeres [11], their significance for nuclear DNA under physiological conditions has been generally dismissed on the grounds of low strand concentration in the cell nucleus [12]. All eukaryotic and prokaryotic genomes are built from double-stranded DNA (dsDNA). In double-stranded genomic DNA, quadruplex structures may be formed using guanines originating either from one strand or from both strands of dsDNA. As first observed by Cao et al [13], the presence of a second strand with a complementary sequence opens the possibility of G-quadruplex configurations in which the four tracts of guanine are distributed between the two strands of DNA. For example, the sequence GGGAGGGACCCACCC is complemented by CCCTCCCTGGGTGGG, and the 12 guanines from both strands may be combined into a single G4 cage structure. The same number of Watson-Crick base pairs need to be broken to create a G4 from this sequence as in the “standard” case of a quadruplex with all guanines coming from the same strand of dsDNA, therefore no major energetic difference between the interstrand and intrastrand configurations should be expected. Nonetheless the definition of a quadruplex-forming sequence used in most genome-wide studies of G-quadruplex DNA is the same as for single-stranded DNA, implicitly assuming that, to form a quadruplex, four tracts of guanine, usually each at least 3nt long, must be positioned in consecutive locations along *the same* strand of DNA. This assumption has become a nearly unchallenged consensus in the field. As a consequence, the sequence motif G_3+_N_1-7_G_3+_N_1-7_G_3+_N_1-7_G_3+_ (or C_3+_N_1-7_C_3+_N_1-7_C_3+_N_1-7_C_3+_ for the complementary strand), see e.g. [14] has been adopted to predict potential sites of G-quadruplex formation in the genome. This motif, or its variants with different limits on the length of the loops, has been used in most algorithms for predicting putative quadruplex sequences (PQS), including *Quadparser* [12], *G4 Calculator* [15], *QGRS Mapper* [16], and others. Likewise, PQS databases, e.g. ([17,18]), and whole-genome analysis studies in higher eukaryotes, e.g. ([5,6,7,10,14,19–28]), amounting to several hundred reports published to date, have used this motif or its variant [29]; with notable exceptions in the papers mapping possible quadruplexes in the yeast genome [13] and the human mitochondrial genome [30].

Here, I use a modified version of the approach of Cao et al. [13] to identify sequences potentially forming G-quadruplexes of all types in the human genome, demonstrating their high prevalence. Analysis of enrichment and overlap with functional sites points to association of distinct types of functional loci with the different topologies of G-quadruplex structures, which may suggest that the different G4 topologies are involved in different cellular processes.

## Materials and Methods

### Prediction of interstrand G-Quadruplexes

Potential quadruplex-forming sequences in the genome have been defined by the regular expression (PCRE type [31]):

- m/(G{3,}).{1,7}\1.{1,7}\1.{1,7}\1|(C{3,}).{1,7}\2.{1,7}\2.{1,7}\2/g

for single-strand PQS, and by the following regular expressions

- m/(G{3,}).{1,7}\1.{0,7}(C{3,}).{1,7}\2/g
- m/(C{3,}).{1,7}\1.{0,7}(G{3,}).{1,7}\2/g
- m/(G{3,}).{0,7}(C{3,}).{1,7}\2.{0,7}\1|(C{3,}).{0,7}(G{3,}).{1,7}\4.{0,7}\3/g
- m/(G{3,}).{0,7}(C{3,}).{0,7}\1.{0,7}\2/g
- m/(C{3,}).{0,7}(G{3,}).{0,7}\1.{0,7}\2/g
- m/(G{3,}).{0,7}(C{3,}).{1,7}\2.{1,7}\2|(G{3,}).{1,7}\3.{1,7}\3.{0,7}(C{3,})/g m/(C{3,}).{0,7}(G{3,}).{1,7}\2.{1,7}\2|(C{3,}).{1,7}\3.{1,7}\3.{0,7}(G{3,})/g m/(G{3,}).{0,7}(C{3,}).{0,7}\1.{1,7}\1|(C{3,}).{1,7}\3.{0,7}(G{3,}).{0,7}\3/g m/(C{3,}).{0,7}(G{3,}).{0,7}\1.{1,7}\1|(G{3,}).{1,7}\3.{0,7}(C{3,}).{0,7}\3/g

for the different topology classes of cross-strand G-quadruplexes. The first regular expression produces results nearly identical to the *Quadparser* software [12], with minor differences due to different implicit heuristics applied in situations where alternative or overlapping PQS sequences exist. Similarly, my approach to finding interstrand quadruplex-forming sequences differs from the approach of Cao et al. [13] in that here a separate one-step search is performed for each topology class, while Cao et al. first identify DNA intervals with quadruplex-forming potential and then characterize the topology of the possible quadruplex. As a result, in certain cases of partially overlapping quadruplex-forming sequences some of the alternative topology types may be missed by the two-step approach, although my one-step method requires additional processing of the results if only non-overlapping sequences are desired. A complete Perl program and the results for the hg19 human genome assembly are available as supplementary data, and from the supporting website http://moment.utmb.edu/allquads (the website also provides the results for the hg18 and hg38 assemblies). The program reads a fasta file and outputs PQS’s in text format, one per line, including sequence id, topology class, position and the PQS sequence. The post-processing required to identify overlapping quadruplex-forming sequences is a straightforward task. It can be implemented as an algorithm with O(N log N) computational complexity in the number of quadruplexes when the PQS and DS-PQS sequences are first sorted according to chromosomal coordinate, this function is implemented among others by the *intersectBed* command of the *bedtools* package [32].

### Analysis of functional associations

Following *Gray et al*. [5], I also predicted the potential PQS and DS-PQS sequences allowing for loops of up to 12 nt between the guanine tracts (replacing 7 with 12 in the regular expressions above), and used them in the functional analysis of PQS and DS-PQS sites compared to sequencing data on transcriptional initiation and origins of replication. The analysis used the hg19 build of the genome, with the exception of the *hf2* antibody pull-down results [19] that have been mapped to hg18. To analyze the prevalence of PQS and DS-PQS sequences in promoter regions, I applied the *allquads*.*pl* algorithm directly to the *upstream1000*.*fa* fasta file obtained from the UCSC genome database on February 17^th^, 2015. To infer functions enriched in the DS-PQS loci, I analyzed their overlaps with experimentally identified sites of transcription initiation and origins of replication. The binomial tests for enrichment were performed as in [5], using the *gsl_cdf_binomial_Q* function included in the *Math*::*GSL*::*CDF CPAN* library [33].

The ChIP-seq, pull-down, G4-seq and nascent DNA sequencing peaks were obtained from GEO accession numbers GSE44849 (GSM1092544, GSM1092545), GSE28911 (GSM716435, GSM716437), GSE63874 and GSE45241 (GSM1099724, GSM1099725, GSM1099726, GSM1099727). Overlaps between peaks and G4’s were defined if there was at least one base pair common to both features.

## Results

### Prevalence of interstrand quadruplex forming sequences

**Fig 1:**
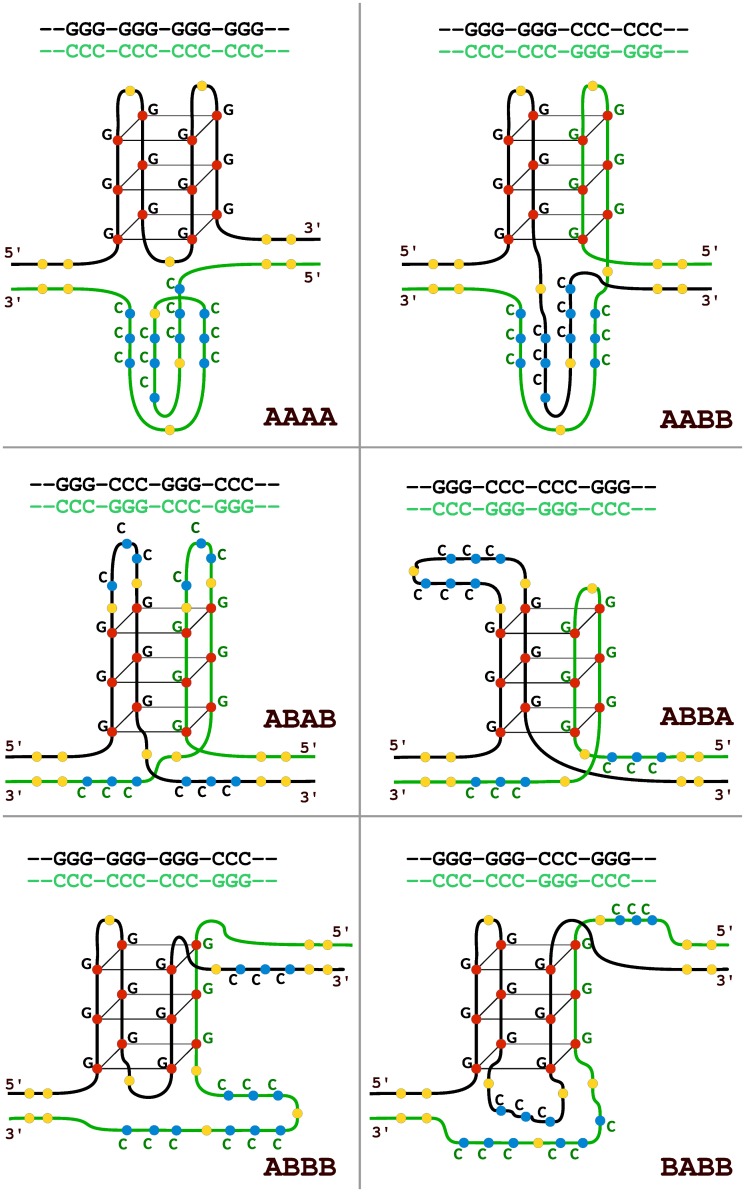
Examples of topology classes of G-quadruplex structures within genomic DNA. Examples of topological configurations of intrastrand and interstrand quadruplexes are shown schematically. For pairs of topology types that differ only by strand from which the sequence is derived (e.g. ABAB and BABA) only one is shown. Black, green—the Watson and Crick strands. Red: guanines, blue: cytosines, yellow: loops of up to seven nucleotides of any type. AAAA—the intrastrand topology, AABB, ABAB, ABBA, ABBB, BABB: interstrand configurations.

Nine classes of G4 topologies involving both DNA strands are possible in addition to the previously described case of all guanines located on one strand of dsDNA [13], or five if one ignores the difference between quadruplex starting from the positive and one starting from the negative strand; example topological configurations are shown in Fig 1. Depending on the order of guanine and cytosine tracts on either strand within the sequence, I will denote them as AABB (4), ABAA (8), ABAB (6), ABBA (7), ABBB (2), BAAA (1), BABA (5), BABB (9) and BBAA (3), numbers in parentheses correspond to pattern classes as defined by Cao et al. [13]. AAAA stands for the widely discussed single-strand configuration; generally “A” represents a guanine tract and “B” a cytosine tract, counting from the 5’ end of either strand, reverse complements are not distinguished (e.g. AABA and BABB are the same). Note that each of the ten classes allows several conformations (differing by polarity and arrangement of loops), however they cannot be distinguished based on sequence alone. It is likely that certain topologies will allow formation of a hybrid *i-motif* [4] in addition to the G4, depending on the lengths of the loops connecting the runs of guanine and cytosine. The i-motif requires a specific range of pH [34,35] and its significance in-vivo is thus limited, therefore although i-motifs in physiological conditions have been reported in certain cases [36,37,38], in this paper I will focus on the G-quadruplex only.

To identify putative double-strand-derived quadruplex sequences (DS-PQS) in the human genome, I implemented an algorithm representing the sequence search in terms of Perl-compatible regular expressions. The source code of the program *allquads*.*pl* is available as a supplementary material (S1 File) as well as from the supporting website *http*:*//moment*.*utmb*.*edu/allquads*. A genome-wide search with loop lengths between 1 and 7 nucleotides (0 to 7 for loops between guanine runs on opposite strands) has revealed 897,935 DS-PQS sequences, of which 196,953 have an overlap of at least one nucleotide with one of the 374,834 single-strand (AAAA) PQS’s. 150,294 DS-PQS are of the BAAA topology class (97,565 without overlap with a single-strand quadruplex), 152,329 (99,299 not overlapping with a single-strand PQS)–ABBB, 69,198 (56,445)–AABB, 142,890 (115,766)–ABAA, 55,735 (50,866)–ABAB, 96,163 (87,940)–ABBA, 49,558 (44,976)–BABA, 128,404 (101,561)–BABB and 53,364 (46,024)–BBAA. Notably, a significant asymmetry exists in the prevalence of some of the ‘mirror image’ configurations: AABB is more abundant than BBAA, ABAB is more abundant than BABA, and ABAA than BABB. Similar differences are present in the yeast genome, see fig. 4a of Cao et al. [13].

Taking into account the overlaps between DS-PQS’s of different topologies, 550,977 independent DS-PQS sites are present in the human genome. Similarly, 832,540 independent groups of overlapping quadruplex-forming sequences of any type (interstrand or intrastrand) are found. The complete list of human DS-PQS sites is available in the online supplement (S2 File) and from the supporting website. The numbers of identified interstrand and intrastrand PQS sites per chromosome are shown in Table 1, and detailed breakdown by topology class is listed in S1 Table. While the average PQS density (per megabase) varies significantly from chromosome to chromosome, the ratios of numbers of intrastrand to interstrand PQS sequences for every chromosome are close to the genome average of 0.68, with the exception of the X and Y chromosomes that are depleted in DS-PQS sites and have intrastrand to interstrand ratios of 0.81 and 0.82 respectively. This statistically significant difference (20.7σ and 9.3σ respectively, Poisson model) may reflect different functions of genes, different regulation, or different chromatin organization in the sex chromosomes compared to autosomes.

**Table 1:** Intrastrand and interstrand G-quadruplex sequences by human chromosome.

Chromosome	Unique interstrand		Intrastrand		Intra/inter
	count	per MB	count	per MB	ratio
**Genome wide**	550,977	177.98	374,834	121.08	0.68
**chr1**	48,860	196.03	32,597	130.78	0.67
**chr2**	39,032	160.49	26,826	110.30	0.69
**chr3**	26,696	134.81	18,839	95.14	0.71
**chr4**	21,369	111.79	15,358	80.34	0.72
**chr5**	24,701	136.53	17,587	97.21	0.71
**chr6**	22,788	133.17	16,675	97.45	0.73
**chr7**	28,578	179.58	19,372	121.73	0.68
**chr8**	22,872	156.27	15,750	107.61	0.69
**chr9**	26,855	190.17	17,522	124.08	0.65
**chr10**	26,533	195.77	17,420	128.53	0.66
**chr11**	30,587	226.56	19,984	148.02	0.65
**chr12**	23,132	172.82	16,330	122.00	0.71
**chr13**	11,244	97.63	7,802	67.74	0.69
**chr14**	17,177	160.01	11,311	105.37	0.66
**chr15**	17,585	171.51	11,733	114.43	0.67
**chr16**	25,607	283.41	16,495	182.56	0.64
**chr17**	29,171	359.27	18,897	232.74	0.65
**chr18**	10,458	133.94	7,474	95.73	0.71
**chr19**	30,206	510.85	20,794	351.67	0.69
**chr20**	17,655	280.12	11,496	182.40	0.65
**chr21**	7,857	163.25	4,848	100.73	0.62
**chr22**	18,280	356.30	10,420	203.10	0.57
**chrX**	20,295	130.71	16,468	106.06	**0.81**
**chrY**	3,436	57.87	2,832	47.70	**0.82**

For each chromosome in the human nuclear genome (v. hg19), the numbers of intrastrand and interstrand potentially quadruplex-forming sequences are listed. Sequences with loops up to 7nt long and at least three guanines per tract are considered. In case of overlapping interstrand quadruplexes, only one is taken into account (unique DS-PQS).

The presented prevalence of potentially quadruplex-forming sequences have been computed for the standard human genome. While the genomes of human cell lines used in research (such as HeLa or HEK293) differ from the standard genome assembly, most of the differences are translocations or copy number variations that do not have significant impact on the presence or absence of sequences potentially forming G-quadruplexes. Local polymorphisms specific to the cell lines are limited to a relatively small number of sequences, and are not expected to affect the global statistics of PQS sites. For example, the polymorphisms specific to the HEK293T line, as identified by [39], do not overlap with any of the quadruplex-forming sites, either interstrand or intrastrand. Therefore, the results can be readily applied to the genomes of research cell lines.

### Functions of sites with potential to form interstrand G4s

The high abundance of DS-PQS sites opens the possibility that interstrand quadruplexes may play a role in a major cellular process. Indirect evidence in favor of such a role may be derived from association between DS-PQS sites and genomic loci with functional properties known to involve G4 structures. Intrastrand G-quadruplexes and G-quadruplex forming sequences have been reported to coincide with promoter regions and play a role in transcriptional regulation [4,28,40–46]. Indeed, at least one single-strand (AAAA) PQS is present within 45.0% regions 1-kb upstream of a transcription start site. Searching for DS-PQS sites reveals potential interstrand quadruplexes in 52.5% of these sequences (p < 10^−308^; binomial); a total of 63.1% of human transcripts have at least one putative G-quadruplex of any type in their 1-kb upstream region.

Additional evidence for the role of G4s in transcription initiation has been provided by a recent ChIP-seq study mapping the binding sites of transcriptional helicases XPB and XPD [5]: approximately 20% of XPB and XPD ChIP-seq peaks overlap with a single-strand PQS (approximately 40% when the PQS definition is relaxed to include loops of up to 12nt connecting the guanine runs). I have analyzed the data in the context of quadruplex—forming sites of all types. The overlaps of XPB and XPD binding sites with all detected G4 sequences—including interstrand—are significantly higher: 45% and 48% respectively for standard loop length of up to 7nt; or 70% and 73% respectively for XPB and XPD when allowing for loops up to 12nt long (see details in Table 2 and S2 Table). This result, along with the enrichment in promoter regions described above, demonstrates a significant association of DS-PQS sites with transcriptional initiation (p < 10^−308^; binomial test for enrichment of DS-PQS both in XPB and in XPD peaks). Notably, the enrichment of DS-PQS’s in transcriptional helicase binding sites is higher than for interstrand PQS’s, and there is no enrichment at all of peaks containing an intrastrand PQS but no DS-PQS; this observation is consistent with XPB and XPD binding only at interstrand G4’s; the enrichment of intrastrand PQS reported by [5] may be explained by intrastrand PQS overlapping with DS-PQS or present in some of the loci containing a DS-PQS.

**Table 2:** Intrastrand and interstrand G-quadruplex forming sequences associated with functional sites in the human genome.

		Intrastrand PQS			DS-PQS				Any PQS			DS/SS
Site	# in genome	with G4	Enr.	%sites	with G4	Enr.	%sites	significance	with G4	Enr.	%sites	ratio
**XPD peaks**	14,570	3,288	1.99	22.6%	6,052	2.49	41.5%	< 1e-308	6,995	1.91	48.0%	1.84
**XPB peaks**	21,555	4,466	2.05	20.7%	8,363	2.61	38.8%	< 1e-308	9,769	2.02	45.3%	1.87
**ORI MCF7**	94,195	24,117	5.05	25.6%	34,425	4.90	36.6%	< 1e-308	42,108	3.97	44.7%	1.43
**ORI K562**	62,971	12,583	5.08	20.0%	16,724	4.59	26.6%	< 1e-308	22,331	4.05	35.5%	1.33
**1kb upstream**	39,927	17,975	3.96	45.0%	20,976	3.15	52.5%	< 1e-308	25,199	2.50	63.1%	1.17
**Hf2 bind (1+)**	9,149	1,095	1.34	12.0%	754	0.63	8.2%	not enriched	1,582	0.87	17.3%	0.69
**Hf2 bind (2+)**	771	177	1.43	23.0%	128	0.70	16.6%	not enriched	256	0.93	33.2%	0.72
**G4seq Pds&K**^**+**^	490,269	177,119	39.8	36.2%	108,229	16.6	22.1%	< 1e-308	226,496	22.4	46.2%	0.61
**Unique G4s**		374,834			550,977				832,540			1.47

For each type of functional site (transcriptional helicase binding, origin of replication, promoter, *hf2* antibody binding), the number and percentage of sites with a G4 motif, and enrichment ratio of G4’s within the sites are listed. Data are provided for intrastrand PQS’s, inter-strand PQS’s, and for PQS of any type, with loops up to 7nt long. For enriched DS-PQS’s, a limit on binomial significance is listed. Rightmost column: ratio of number of sites with DS-PQS to number of sites with intrastrand PQS; for XPD and XPB binding sites it exceeds the whole-genome ratio (bottom row).

G4 structures have also been associated with origins of replication. To investigate whether this also applies to cross-strand topologies, I considered the overlap between DS-PQS’s and origins of replication that have been mapped by sequencing short nascent DNA in human MCF7 and K562 cells [47]. Again, while single-strand PQS’s are significantly enriched in the replication origins (present respectively in 20% and 26% of the peaks in K562 and MCF7 libraries), DS-PQS’s are even more prevalent (25% and 35%, p < 10^−308^ in both cases), resulting in 34% and 44% origins respectively overlapping with at least one G4 of any type, see Table 1.

In a recent study, a *hf2* antibody that binds to G4 but not to dsDNA was used in a genome-wide pull-down experiment to characterize stable G4 structures in the human genome [19], revealing that 12% of the *hf2* binding sites overlap with an intrastrand PQS. In *hf2* peaks common to two or more replicate experiments, the ratio is 23%. When both intrastrand and interstrand PQS’s are taken into account, up to 17% of *hf2* peaks (33% for two or more libraries) are associated with a potential quadruplex sequence. In this dataset, the ratio of interstrand to intrastrand G4’s is lower than in the functional studies above, and there is no significant enrichment of DS-PQS’s in the sequencing peaks. This result does not however contradict previous findings because the *hf2* antibody was designed and tested for specificity only to intramolecular G-quadruplexes, derived from a single strand of DNA.

The resulting low ratio of interstrand to intrastrand quadruplexes is similar in the more sensitive *G4-seq* study of Chambers et al. [48], who detect quadruplexes by analysing sequencing mismatches between conditions promoting and disfavouring G4 formation. The *G4-seq* approach to quadruplex detection involves separate analysis for each strand of DNA and thus appears to favour intrastrand quadruplexes. Nonetheless 108,229 quadruplex sites overlap with DS-PQS loci (16-fold enriched in DS-PQS sequences), including 49,377 observed interstrand quadruplexes not overlapping with an intrastrand PQS, corresponding to a very significant 9.1-fold enrichment (this calculation is based on quadruplexes observed by [48] simultaneously in the K^+^ and the PDS experiments). The enrichment provides evidence that the G4-seq method does detect DS-PQS sites that do not coincide with an intrastrand PQS.

### Enrichment of topology classes among DS-PQS associated with different functional loci

While the structures of interstrand G4s with different topologies are yet to be determined crystallographically, structural differences between them may be significant for the specific functions of the quadruplex structures. Specifically, if interstrand quadruplexes are functional in transcription initiation or in replication origin, different ratios of numbers of PQS’s with particular topology classes may be expected in the quadruplex-forming sequences associated with such functional elements. The numbers of potentially quadruplex-forming sequences in each topological category, associated with each type of functional element are listed in S3 Table, along with the fractions of all PQS’s and all DS-PQS’s that they constitute, and a comparison with the ratios computed genome-wide, irrespective of functional site. The enrichment calculation uses all predicted quadruplex-forming structures with a 7nt limit on loop length, including overlapping PQS’s with different topologies, as any of the overlapping PQS’s can be potentially functional. Generally, among the DS-PQS’s coinciding with origin of replication sites, the BABB, ABBB and BAAA topologies are significantly enriched (between 5σ and 24σ; asymptotic estimation for Poisson distribution), compared to the genome-wide prevalence. In the transcriptional helicase binding sites, the ABBA, BABB and BBAA topologies are enriched compared to their genome-wide abundances, while the intrastrand AAAA is consistently very strongly depleted (>18 σ). Interestingly, while the some of the “mirrored” topologies have different abundances genome-wide (e.g. AABB vs. BBAA, or ABAA vs. BABB), their abundances in many functional sites are nearly equal, suggesting different mechanism of selection of quadruplex topologies in functional and non-functional loci. Generally, these results constitute evidence of functional preference of quadruplex-forming sequences of different topology classes, and suggest that the function depends on the topology and structure of the interstrand G-quadruplex formed within the genomic DNA.

## Discussion

By integrated sequence-based prediction with results of functional studies, I have shown that sequences potentially forming interstrand G-Quadruplexes, a nucleic acid structure previously not considered in higher eukaryotic nuclear DNA, are highly prevalent in the human genome and colocalize with functionally significant loci. Enrichments of interstrand and intrastrand PQS’s in the functional studies suggest that in DNA replication interstrand G4 conformations may have serve a function similar to intrastrand quadruplexes. In transcription initiation, the role of DS-PQS is, in the light of this analysis, even more prominent than that of intrastrand quadruplexes; possibly only interstrand G-quadruplexes are involved in recruitment of transcriptional helicases. Both single-strand and double-strand PQS’s should be considered in future studies of these and other functions of G4s in the nuclear DNA.

## Supporting Information

S1 File Source code of the AllQuads program for predicting interstrand G4-forming sequences. (TAR)

S2 File The complete list of interstrand and intrastrand quadruplex sites in the human genome (hg19), with at least three guanines per tract and loops not longer than 7nt–(tar archive of compressed text files, one per chromosome). (TAR)

S1 Table Abundance of intrastrand and interstrand G-quadruplex sequences of different topology classes in human chromosomes (separate pdf file). (PDF)

S2 Table Detailed functional analysis of intrastrand and interstrand G-quadruplex sequences in human genome (separate pdf file). (PDF)

S3 Table Relative abundances of PQS’s of different topology classes in the whole genome, and in the functional regions, calculated for all PQS’s and for interstrand PQS only. XPD, XPB—transcriptional helicase binding sites; ORI–origins of replication; hf2 –antibody to intrastrand PQS, upstream1000 –promoter regions. Enrichments of ratios in functional sites compared to genome-wide proportions of PQS’s with different topology classes suggest that different PQS topologies may be responsible for different functions. Bottom panels—z-transformed enrichments, compared to genome-wide ratios for all PQS’s and for interstrand PQS only; negative numbers denote depletion. (separate pdf file).
(PDF)
