# Supplementary material for: G-Quadruplexes Involving Both Strands of Genomic DNA Are Highly Abundant and Colocalize with Functional Sites in the Human Genome
Source: PLoS One. 2016 Jan 4;11(1):e0146174. doi: 10.1371/journal.pone.0146174 (PMC4699641; doi:10.1371/journal.pone.0146174)
Supplement: S1 Table — (PDF) [file pone.0146174.s003.pdf]

Topology	AAAA			AABB			ABAA			ABAB		
Chromosome	count	fraction all	%WG	count	fraction all	%WG	count	fraction all	%WG	count	fraction all	%WG
Genome wide	374,834	0.2945 +/- 0.0005	100.0%	69,198	0.0544 +/- 0.0002	100.0%	142,890	0.1123 +/- 0.0003	100.0%	55,735	0.0438 +/- 0.0002	100.0%
chr1	32,597	0.2890 +/- 0.0016	98.1%	6,225	0.0552 +/- 0.0007	101.5%	12,311	0.1092 +/- 0.0010	97.2%	4,864	0.0431 +/- 0.0006	98.5%
chr2	26,826	0.3039 +/- 0.0019	103.2%	4,867	0.0551 +/- 0.0008	101.4%	9,867	0.1118 +/- 0.0011	99.6%	3,814	0.0432 +/- 0.0007	98.7%
chr3	18,839	0.3162 +/- 0.0023	107.4%	3,389	0.0569 +/- 0.0010	104.6%	6,685	0.1122 +/- 0.0014	99.9%	2,524	0.0424 +/- 0.0008	96.7%
chr4	15,358	0.3179 +/- 0.0026	107.9%	2,750	0.0569 +/- 0.0011	104.7%	5,851	0.1211 +/- 0.0016	107.9%	2,254	0.0467 +/- 0.0010	106.5%
chr5	17,587	0.3175 +/- 0.0024	107.8%	3,131	0.0565 +/- 0.0010	104.0%	6,414	0.1158 +/- 0.0014	103.1%	2,448	0.0442 +/- 0.0009	100.9%
chr6	16,675	0.3226 +/- 0.0025	109.5%	2,884	0.0558 +/- 0.0010	102.6%	5,679	0.1099 +/- 0.0015	97.9%	2,258	0.0437 +/- 0.0009	99.8%
chr7	19,372	0.2932 +/- 0.0021	99.5%	3,718	0.0563 +/- 0.0009	103.5%	7,401	0.1120 +/- 0.0013	99.8%	2,839	0.0430 +/- 0.0008	98.1%
chr8	15,750	0.3056 +/- 0.0024	103.8%	2,743	0.0532 +/- 0.0010	97.9%	6,002	0.1165 +/- 0.0015	103.7%	2,351	0.0456 +/- 0.0009	104.2%
chr9	17,522	0.2797 +/- 0.0021	95.0%	3,385	0.0540 +/- 0.0009	99.4%	7,133	0.1138 +/- 0.0013	101.4%	2,754	0.0440 +/- 0.0008	100.4%
chr10	17,420	0.2899 +/- 0.0022	98.4%	3,139	0.0522 +/- 0.0009	96.1%	6,754	0.1124 +/- 0.0014	100.1%	2,691	0.0448 +/- 0.0009	102.3%
chr11	19,984	0.2831 +/- 0.0020	96.1%	3,966	0.0562 +/- 0.0009	103.4%	7,883	0.1117 +/- 0.0013	99.5%	3,111	0.0441 +/- 0.0008	100.7%
chr12	16,330	0.3030 +/- 0.0024	102.9%	2,947	0.0547 +/- 0.0010	100.6%	6,049	0.1122 +/- 0.0014	100.0%	2,391	0.0444 +/- 0.0009	101.3%
chr13	7,802	0.3082 +/- 0.0035	104.6%	1,406	0.0555 +/- 0.0015	102.1%	2,962	0.1170 +/- 0.0021	104.2%	1,174	0.0464 +/- 0.0014	105.9%
chr14	11,311	0.2886 +/- 0.0027	98.0%	2,090	0.0533 +/- 0.0012	98.1%	4,422	0.1128 +/- 0.0017	100.5%	1,692	0.0432 +/- 0.0010	98.6%
chr15	11,733	0.2919 +/- 0.0027	99.1%	2,145	0.0534 +/- 0.0012	98.2%	4,352	0.1083 +/- 0.0016	96.4%	1,734	0.0431 +/- 0.0010	98.5%
chr16	16,495	0.2740 +/- 0.0021	93.0%	3,095	0.0514 +/- 0.0009	94.6%	6,683	0.1110 +/- 0.0014	98.9%	2,654	0.0441 +/- 0.0009	100.7%
chr17	18,897	0.2729 +/- 0.0020	92.7%	3,696	0.0534 +/- 0.0009	98.2%	7,253	0.1048 +/- 0.0012	93.3%	2,935	0.0424 +/- 0.0008	96.8%
chr18	7,474	0.3150 +/- 0.0036	107.0%	1,167	0.0492 +/- 0.0014	90.5%	2,735	0.1153 +/- 0.0022	102.7%	1,012	0.0427 +/- 0.0013	97.4%
chr19	20,794	0.2722 +/- 0.0019	92.4%	4,053	0.0531 +/- 0.0008	97.6%	8,279	0.1084 +/- 0.0012	96.5%	3,104	0.0406 +/- 0.0007	92.8%
chr20	11,496	0.2770 +/- 0.0026	94.1%	2,234	0.0538 +/- 0.0011	99.0%	4,511	0.1087 +/- 0.0016	96.8%	1,814	0.0437 +/- 0.0010	99.8%
chr21	4,848	0.2656 +/- 0.0038	90.2%	920	0.0504 +/- 0.0017	92.7%	2,077	0.1138 +/- 0.0025	101.3%	870	0.0477 +/- 0.0016	108.8%
chr22	10,420	0.2452 +/- 0.0024	83.2%	2,204	0.0519 +/- 0.0011	95.4%	4,822	0.1134 +/- 0.0016	101.1%	1,942	0.0457 +/- 0.0010	104.3%
chrX	16,468	0.3506 +/- 0.0027	**119.1%**	2,654	0.0565 +/- 0.0011	103.9%	5,715	0.1217 +/- 0.0016	108.4%	2,081	0.0443 +/- 0.0010	101.2%
chrY	2,832	0.3373 +/- 0.0063	**114.5%**	390	0.0464 +/- 0.0024	85.4%	1,048	0.1248 +/- 0.0039	111.2%	424	0.0505 +/- 0.0025	115.3%

Topology	ABBA			ABBB			BAAA		
Chromosome	count	fraction all	%WG	count	fraction all	%WG	count	fraction all	%WG
Genome wide	96,163	0.0756 +/- 0.0002	100.0%	152,329	0.1197 +/- 0.0003	100.0%	150,294	0.1181 +/- 0.0003	100.0%
chr1	8,571	0.0760 +/- 0.0008	100.6%	13,793	0.1223 +/- 0.0010	102.2%	13,863	0.1229 +/- 0.0010	104.1%
chr2	6,477	0.0734 +/- 0.0009	97.1%	10,413	0.1180 +/- 0.0012	98.6%	10,357	0.1173 +/- 0.0012	99.4%
chr3	4,345	0.0729 +/- 0.0011	96.5%	6,832	0.1147 +/- 0.0014	95.8%	6,798	0.1141 +/- 0.0014	96.6%
chr4	3,457	0.0716 +/- 0.0012	94.7%	5,386	0.1115 +/- 0.0015	93.2%	5,081	0.1052 +/- 0.0015	89.1%
chr5	3,889	0.0702 +/- 0.0011	92.9%	6,223	0.1123 +/- 0.0014	93.9%	6,306	0.1138 +/- 0.0014	96.4%
chr6	3,545	0.0686 +/- 0.0012	90.8%	6,089	0.1178 +/- 0.0015	98.4%	5,820	0.1126 +/- 0.0015	95.3%
chr7	5,131	0.0777 +/- 0.0011	102.8%	7,964	0.1205 +/- 0.0014	100.7%	7,644	0.1157 +/- 0.0013	98.0%
chr8	3,695	0.0717 +/- 0.0012	94.9%	5,977	0.1160 +/- 0.0015	96.9%	5,818	0.1129 +/- 0.0015	95.6%
chr9	4,876	0.0778 +/- 0.0011	103.0%	7,624	0.1217 +/- 0.0014	101.7%	7,554	0.1206 +/- 0.0014	102.1%
chr10	4,657	0.0775 +/- 0.0011	102.6%	7,151	0.1190 +/- 0.0014	99.4%	7,051	0.1174 +/- 0.0014	99.4%
chr11	5,428	0.0769 +/- 0.0010	101.8%	8,564	0.1213 +/- 0.0013	101.4%	8,543	0.1210 +/- 0.0013	102.5%
chr12	3,902	0.0724 +/- 0.0012	95.8%	6,441	0.1195 +/- 0.0015	99.8%	6,172	0.1145 +/- 0.0015	97.0%
chr13	1,862	0.0735 +/- 0.0017	97.3%	2,744	0.1084 +/- 0.0021	90.6%	2,765	0.1092 +/- 0.0021	92.5%
chr14	3,056	0.0780 +/- 0.0014	103.2%	4,632	0.1182 +/- 0.0017	98.8%	4,747	0.1211 +/- 0.0018	102.6%
chr15	3,029	0.0754 +/- 0.0014	99.7%	4,916	0.1223 +/- 0.0017	102.2%	4,903	0.1220 +/- 0.0017	103.3%
chr16	4,932	0.0819 +/- 0.0012	108.4%	7,241	0.1203 +/- 0.0014	100.5%	7,414	0.1231 +/- 0.0014	104.3%
chr17	5,450	0.0787 +/- 0.0011	104.2%	9,016	0.1302 +/- 0.0014	108.8%	8,714	0.1259 +/- 0.0013	106.6%
chr18	1,758	0.0741 +/- 0.0018	98.1%	2,662	0.1122 +/- 0.0022	93.8%	2,728	0.1150 +/- 0.0022	97.4%
chr19	5,937	0.0777 +/- 0.0010	102.9%	10,102	0.1322 +/- 0.0013	110.5%	9,643	0.1262 +/- 0.0013	106.9%
chr20	3,286	0.0792 +/- 0.0014	104.8%	5,227	0.1260 +/- 0.0017	105.2%	5,052	0.1217 +/- 0.0017	103.1%
chr21	1,545	0.0846 +/- 0.0022	112.0%	2,158	0.1182 +/- 0.0025	98.8%	2,123	0.1163 +/- 0.0025	98.5%
chr22	3,699	0.0870 +/- 0.0014	115.2%	5,508	0.1296 +/- 0.0017	108.3%	5,286	0.1244 +/- 0.0017	105.3%
chrX	3,079	0.0656 +/- 0.0012	86.8%	4,864	0.1036 +/- 0.0015	86.5%	4,992	0.1063 +/- 0.0015	90.0%
chrY	557	0.0663 +/- 0.0028	87.8%	801	0.0954 +/- 0.0034	79.7%	918	0.1093 +/- 0.0036	92.6%

Topology	BABA			BABB			BBAA		
Chromosome	count	fraction all	%WG	count	fraction all	%WG	count	fraction all	%WG
Genome wide	49,558	0.0389 +/- 0.0002	100.0%	128,404	0.1009 +/- 0.0003	100.0%	53,364	0.0419 +/- 0.0002	100.0%
chr1	4,341	0.0385 +/- 0.0006	98.9%	11,371	0.1008 +/- 0.0009	99.9%	4,846	0.0430 +/- 0.0006	102.5%
chr2	3,418	0.0387 +/- 0.0007	99.4%	8,607	0.0975 +/- 0.0011	96.7%	3,625	0.0411 +/- 0.0007	97.9%
chr3	2,148	0.0360 +/- 0.0008	92.6%	5,668	0.0951 +/- 0.0013	94.3%	2,360	0.0396 +/- 0.0008	94.5%
chr4	1,715	0.0355 +/- 0.0009	91.2%	4,647	0.0962 +/- 0.0014	95.3%	1,811	0.0375 +/- 0.0009	89.4%
chr5	2,032	0.0367 +/- 0.0008	94.2%	5,210	0.0940 +/- 0.0013	93.2%	2,157	0.0389 +/- 0.0008	92.9%
chr6	1,902	0.0368 +/- 0.0008	94.5%	4,760	0.0921 +/- 0.0013	91.3%	2,079	0.0402 +/- 0.0009	95.9%
chr7	2,617	0.0396 +/- 0.0008	101.7%	6,684	0.1012 +/- 0.0012	100.3%	2,708	0.0410 +/- 0.0008	97.7%
chr8	2,071	0.0402 +/- 0.0009	103.2%	5,067	0.0983 +/- 0.0014	97.4%	2,067	0.0401 +/- 0.0009	95.7%
chr9	2,590	0.0413 +/- 0.0008	106.2%	6,438	0.1028 +/- 0.0013	101.9%	2,777	0.0443 +/- 0.0008	105.7%
chr10	2,442	0.0406 +/- 0.0008	104.4%	6,249	0.1040 +/- 0.0013	103.1%	2,531	0.0421 +/- 0.0008	100.5%
chr11	2,710	0.0384 +/- 0.0007	98.6%	7,359	0.1043 +/- 0.0012	103.3%	3,034	0.0430 +/- 0.0008	102.5%
chr12	2,032	0.0377 +/- 0.0008	96.8%	5,381	0.0998 +/- 0.0014	99.0%	2,254	0.0418 +/- 0.0009	99.7%
chr13	944	0.0373 +/- 0.0012	95.8%	2,576	0.1017 +/- 0.0020	100.9%	1,082	0.0427 +/- 0.0013	101.9%
chr14	1,574	0.0402 +/- 0.0010	103.1%	3,943	0.1006 +/- 0.0016	99.7%	1,725	0.0440 +/- 0.0011	105.0%
chr15	1,546	0.0385 +/- 0.0010	98.8%	4,177	0.1039 +/- 0.0016	103.0%	1,657	0.0412 +/- 0.0010	98.3%
chr16	2,504	0.0416 +/- 0.0008	106.8%	6,466	0.1074 +/- 0.0013	106.5%	2,721	0.0452 +/- 0.0009	107.8%
chr17	2,770	0.0400 +/- 0.0008	102.8%	7,410	0.1070 +/- 0.0012	106.1%	3,093	0.0447 +/- 0.0008	106.6%
chr18	909	0.0383 +/- 0.0013	98.4%	2,326	0.0980 +/- 0.0020	97.2%	953	0.0402 +/- 0.0013	95.8%
chr19	2,996	0.0392 +/- 0.0007	100.7%	8,184	0.1071 +/- 0.0012	106.2%	3,299	0.0432 +/- 0.0008	103.0%
chr20	1,774	0.0427 +/- 0.0010	109.8%	4,405	0.1061 +/- 0.0016	105.2%	1,701	0.0410 +/- 0.0010	97.8%
chr21	831	0.0455 +/- 0.0016	116.9%	2,047	0.1121 +/- 0.0025	111.1%	837	0.0458 +/- 0.0016	109.4%
chr22	1,874	0.0441 +/- 0.0010	113.2%	4,751	0.1118 +/- 0.0016	110.8%	1,998	0.0470 +/- 0.0011	112.1%
chrX	1,498	0.0319 +/- 0.0008	81.9%	3,883	0.0827 +/- 0.0013	81.9%	1,735	0.0369 +/- 0.0009	88.1%
chrY	320	0.0381 +/- 0.0021	97.9%	793	0.0944 +/- 0.0034	93.6%	314	0.0374 +/- 0.0021	89.2%
