# Supplementary material for: G-Quadruplexes Involving Both Strands of Genomic DNA Are Highly Abundant and Colocalize with Functional Sites in the Human Genome
Source: PLoS One. 2016 Jan 4;11(1):e0146174. doi: 10.1371/journal.pone.0146174 (PMC4699641; doi:10.1371/journal.pone.0146174)
Supplement: S2 Table — (PDF) [file pone.0146174.s004.pdf]

Site		tot. length	% genome	# total	Intrastrand PQS # sites	%G4's	enr.	% sites	DS-PQS # sites	%G4's	enr.	% sites	Any PQS #sites	%G4's	enr.	% sites	SS PQS only #sites	%G4's	enr.	DS PQS only #sites	%G4's	enr.	DS/SS ratio
1 kb up	1kb upstr	39927000	1.21%	39927	17975	4.80%	3.96	45.0%	20976	3.81%	3.15	52.5%	25199	3.03%	2.50	63.1%	4223	1.50%	1.24	7224	1.58%	1.30	1.17
Hf2 antibody	1+ libraries	7177070	0.22%	9149	1095	0.29%	1.34	12.0%	754	0.14%	0.63	8.2%	1582	0.19%	0.87	17.3%	828	0.29%	1.35	487	0.11%	0.49	0.69
	2+ libraries	1088783	0.033%	771	177	0.05%	1.43	23.0%	128	0.02%	0.70	16.6%	256	0.031%	0.93	33.2%	128	0.05%	1.38	79	0.02%	0.52	0.72
G4-seq	PDS & K+	39141070	1.19%	490269	177119	47.25%	39.84	36.1%	108229	19.64%	16.56	22.1%	226496	27.2%	22.94	46.2%	118267	42.0%	35.41	49377	10.8%	9.10	0.61
XPD peaks	0nt–7nt loop	14539969	0.44%	14570	3288	0.88%	1.99	22.6%	6052	1.10%	2.49	41.5%	6995	0.84%	1.91	48.0%	943	0.33%	0.76	3707	0.81%	1.84	1.84
	0nt–12nt loop	14539969	0.44%	14570	5852	1.56%	3.54	40.2%	9984	1.81%	4.11	68.5%	10665	1.28%	2.91	73.2%	681	0.24%	0.55	4813	1.05%	2.39	1.71
XPB peaks	0nt–7nt loop	19179584	0.58%	21555	4466	1.19%	2.05	20.7%	8363	1.52%	2.61	38.8%	9769	1.17%	2.02	45.3%	1406	0.50%	0.86	5303	1.16%	1.99	1.87
	0nt–12nt loop	19179584	0.58%	21555	8146	2.17%	3.74	37.8%	14100	2.56%	4.40	65.4%	15154	1.82%	3.13	70.3%	1054	0.37%	0.64	7008	1.53%	2.63	1.73
ORI MCF7	0nt–7nt loop	42049150	1.27%	94195	24117	6.43%	5.05	25.6%	34425	6.25%	4.90	36.5%	42108	5.06%	3.97	44.7%	7683	2.73%	2.14	17991	3.93%	3.08	1.43
	0nt–12nt loop	42049150	1.27%	94195	39781	10.61%	8.33	42.2%	55157	10.01%	7.86	58.6%	62288	7.48%	5.87	66.1%	7131	2.53%	1.99	22507	4.92%	3.86	1.39
ORI K562	0nt–7nt loop	21824174	0.66%	62971	12583	3.36%	5.08	20.0%	16724	3.04%	4.59	26.6%	22331	2.68%	4.06	35.5%	5607	1.99%	3.01	9748	2.13%	3.22	1.33
	0nt–12nt loop	21824174	0.66%	62971	23208	6.19%	9.36	36.9%	30140	5.47%	8.27	47.9%	36467	4.38%	6.62	57.9%	6327	2.25%	3.40	13259	2.90%	4.38	1.30
WG unique					374834				550977				832540				281563			457706			1.47

Table S2. Detailed functional analysis of intrastrand and interstrand G-quadruplex sequences in human genome.

Site: type of functional site (transcriptional helicase binding, origin of replication, promoter, hf2 antibody binding)
For helicase peaks and origins of replication, G4 PQS's allowing loops up to 12nt are also shown.
Total length, % genome, # total – number of sites and fraction of genome, regardless of PQS presence
Intrastrand PQS – sites overlapping with a single-strand G4 sequence
DS-PQS – sites overlapping with an interstrand G4
Any PQS – sites overlapping with at least one G4 of any type
SS PQS only – sites overlapping with an intrastrand PQS but not with a DS-PQS
DS PQS only – sites overlapping with an interstrand PQS but not with an intrastand G4
DS/SS – ratio of sites of the given type with at least one interstrand G4 to sites with at least one intrastrand G4

In each category: the number (#sites) and percentage (%sites) of sites with a G4 motif, and enrichment ratio (wrt whole-genome) of G4's within in the sites are listed.
%G4s – fraction of all G4s of this class that are in this type of site.
