# Supplementary material for: G-Quadruplexes Involving Both Strands of Genomic DNA Are Highly Abundant and Colocalize with Functional Sites in the Human Genome
Source: PLoS One. 2016 Jan 4;11(1):e0146174. doi: 10.1371/journal.pone.0146174 (PMC4699641; doi:10.1371/journal.pone.0146174)
Supplement: S3 Table — XPD, XPB—transcriptional helicase binding sites; ORI–origins of replication; hf2 –antibody to intrastrand PQS, upstream1000 –promoter regions. Enrichments of ratios in functional sites compared to genome-wide proportions of PQS’s with different topology classes suggest that different PQS topologies may be responsible for different functions. Bottom panels—z-transformed enrichments, compared to genome-wide ratios for all PQS’s and for interstrand PQS only; negative numbers denote depletion. (separate pdf file). (PDF) [file pone.0146174.s005.pdf]

PQS sequences of different topologies associated with functional sites

PQS by topology and function

Topology	Genome	XPD	XPB	MCF7_ORI	K562_ORI	hf2	upstream1000
AAAA	374834	4338	5809	36594	17533	2174	34903
AABB	69198	1058	1417	6892	2167	102	5468
ABAA	142890	2385	3179	16937	6303	246	13149
ABAB	55735	881	1227	6008	1978	65	4708
ABBA	96163	1838	2445	10938	3518	165	8914
ABBB	152329	2768	3679	20139	7340	289	15371
BAAA	150294	2806	3684	19049	6994	256	16388
BABA	49558	913	1263	5781	1802	89	4944
BABB	128404	2417	3268	17316	6491	267	13494
BBAA	53364	1045	1397	5875	1820	91	5697
ALL	1272769	20449	27368	145529	55946	3744	123036
interstrand	897935	16111	21559	108935	38413	1570	88133

Fraction of all quadruplexes [%]

Topology	Genome	XPD	XPB	MCF7_ORI	K562_ORI	hf2	upstream1000
AAAA	29.45%	21.21%	21.23%	25.15%	31.34%	58.07%	28.37%
AABB	5.44%	5.17%	5.18%	4.74%	3.87%	2.72%	4.44%
ABAA	11.23%	11.66%	11.62%	11.64%	11.27%	6.57%	10.69%
ABAB	4.38%	4.31%	4.48%	4.13%	3.54%	1.74%	3.83%
ABBA	7.56%	8.99%	8.93%	7.52%	6.29%	4.41%	7.25%
ABBB	11.97%	13.54%	13.44%	13.84%	13.12%	7.72%	12.49%
BAAA	11.81%	13.72%	13.46%	13.09%	12.50%	6.84%	13.32%
BABA	3.89%	4.46%	4.61%	3.97%	3.22%	2.38%	4.02%
BABB	10.09%	11.82%	11.94%	11.90%	11.60%	7.13%	10.97%
BBAA	4.19%	5.11%	5.10%	4.04%	3.25%	2.43%	4.63%

Fraction of all PQS in functional categories, compared to genome-wide fractions

Topology	Genome	XPD	XPB	MCF7_ORI	K562_ORI	hf2	upstream1000
AAAA	100.00%	72.03%	72.07%	85.38%	106.41%	197.17%	96.33%
AABB	100.00%	95.16%	95.23%	87.11%	71.24%	50.11%	81.74%
ABAA	100.00%	103.89%	103.47%	103.67%	100.35%	58.53%	95.19%
ABAB	100.00%	98.38%	102.38%	94.28%	80.74%	39.65%	87.38%
ABBA	100.00%	118.96%	118.24%	99.48%	83.23%	58.33%	95.89%
ABBB	100.00%	113.10%	112.32%	115.63%	109.62%	64.50%	104.38%
BAAA	100.00%	116.20%	113.99%	110.85%	105.87%	57.90%	112.80%
BABA	100.00%	114.67%	118.52%	102.02%	82.72%	61.05%	103.20%
BABB	100.00%	117.16%	118.36%	117.94%	115.00%	70.69%	108.71%
BBAA	100.00%	121.88%	121.75%	96.29%	77.59%	57.97%	110.44%

Fraction of Interstrand [%]

AABB	7.71%	6.57%	6.57%	6.33%	5.64%	6.50%	6.20%
ABAA	15.91%	14.80%	14.75%	15.55%	16.41%	15.67%	14.92%
ABAB	6.21%	5.47%	5.69%	5.52%	5.15%	4.14%	5.34%
ABBA	10.71%	11.41%	11.34%	10.04%	9.16%	10.51%	10.11%
ABBB	16.96%	17.18%	17.06%	18.49%	19.11%	18.41%	17.44%
BAAA	16.74%	17.42%	17.09%	17.49%	18.21%	16.31%	18.59%
BABA	5.52%	5.67%	5.86%	5.31%	4.69%	5.67%	5.61%
BABB	14.30%	15.00%	15.16%	15.90%	16.90%	17.01%	15.31%
BBAA	5.94%	6.49%	6.48%	5.39%	4.74%	5.80%	6.46%

Fraction of interstrand G4s in functional categories, compared to genome-wide fractions

AABB	100.00%	85.21%	85.29%	82.10%	73.20%	84.30%	80.51%
ABAA	100.00%	93.03%	92.66%	97.70%	103.11%	98.46%	93.76%
ABAB	100.00%	88.10%	91.69%	88.85%	82.96%	66.70%	86.06%
ABBA	100.00%	106.53%	105.90%	93.76%	85.52%	98.13%	94.44%
ABBB	100.00%	101.28%	100.59%	108.98%	112.64%	108.51%	102.81%
BAAA	100.00%	104.06%	102.09%	104.47%	108.78%	97.42%	111.09%
BABA	100.00%	102.68%	106.15%	96.15%	85.00%	102.71%	101.64%
BABB	100.00%	104.91%	106.00%	111.16%	118.17%	118.93%	107.07%
BBAA	100.00%	109.14%	109.03%	90.75%	79.72%	97.53%	108.77%

Estimated significance of enrichment wrt genome-wide ratios (expressed in standard deviations) – all PQS

AAAA		-18.4	-21.3	-28.0	8.5	45.3	-6.9
AABB		-1.6	-1.8	-10.7	-13.4	-5.0	-13.5
ABAA		1.9	2.0	4.8	0.3	-6.5	-5.5
ABAB		-0.5	0.8	-4.4	-8.6	-4.9	-8.7
ABBA		8.1	9.0	-0.5	-9.9	-5.4	-3.9
ABBB		6.9	7.5	22.2	8.2	-6.0	5.4
BAAA		8.6	8.5	15.0	4.9	-6.7	16.4
BABA		4.4	6.6	1.5	-7.3	-3.7	2.3
BABB		8.4	10.5	23.6	12.1	-4.8	10.1
BBAA		7.1	8.1	-2.8	-9.6	-4.0	7.9

Estimated significance of enrichment wrt genome-wide ratios (expressed in standard deviations) – DS-PQS only

AABB		-4.8	-5.5	-14.9	-12.5	-1.6	-14.4
ABAA		-3.4	-4.1	-3.0	2.5	-0.2	-7.2
ABAB		-3.5	-2.9	-8.6	-7.6	-2.7	-9.6
ABBA		2.8	2.9	-6.5	-8.6	-0.2	-5.2
ABBB		0.7	0.4	12.7	10.8	1.4	3.5
BAAA		2.1	1.3	6.2	7.3	-0.4	14.2
BABA		0.8	2.2	-2.9	-6.4	0.3	1.2
BABB		2.4	3.4	14.7	14.6	3.1	8.2
BBAA		3.0	3.4	-7.1	-8.6	-0.2	6.6
